# Local Ancestry Adjusted Allelic Association Analysis Robustly Captures Tuberculosis Susceptibility Loci

**DOI:** 10.3389/fgene.2021.716558

**Published:** 2021-10-15

**Authors:** Yolandi Swart, Caitlin Uren, Paul D. van Helden, Eileen G. Hoal, Marlo Möller

**Affiliations:** ^1^ DSI-NRF Centre of Excellence for Biomedical Tuberculosis Research, South African Medical Research Council Centre for Tuberculosis Research, Division of Molecular Biology and Human Genetics, Faculty of Medicine and Health Sciences, Stellenbosch University, Cape Town, South Africa; ^2^ Centre for Bioinformatics and Computational Biology, Stellenbosch University, Stellenbosch, South Africa

**Keywords:** South Africa, admixture mapping, TB susceptibility, ancestry-specific risk alleles, local ancestry adjustments, population genetics, host genetics

## Abstract

Pulmonary tuberculosis (TB), caused by *Mycobacterium tuberculosis*, is a complex disease. The risk of developing active TB is in part determined by host genetic factors. Most genetic studies investigating TB susceptibility fail to replicate association signals particularly across diverse populations. South African populations arose because of multi-wave genetic admixture from the indigenous KhoeSan, Bantu-speaking Africans, Europeans, Southeast Asian-and East Asian populations. This has led to complex genetic admixture with heterogenous patterns of linkage disequilibrium and associated traits. As a result, precise estimation of both global and local ancestry is required to prevent both false positive and false-negative associations. Here, 820 individuals from South Africa were genotyped on the SNP-dense Illumina Multi-Ethnic Genotyping Array (∼1.7M SNPs) followed by local and global ancestry inference using RFMix. Local ancestry adjusted allelic association (LAAA) models were utilized owing to the extensive genetic heterogeneity present in this population. Hence, an interaction term, comprising the identification of the minor allele that corresponds to the ancestry present at the specific locus under investigation, was included as a covariate. One SNP (rs28647531) located on chromosome 4q22 was significantly associated with TB susceptibility and displayed a SNP minor allelic effect (G allele, frequency = 0.204) whilst correcting for local ancestry for Bantu-speaking African ancestry (*p*-value = 5.518 × 10^−7^; OR = 3.065; SE = 0.224). Although no other variants passed the significant threshold, clear differences were observed between the lead variants identified for each ancestry. Furthermore, the LAAA model robustly captured the source of association signals in multi-way admixed individuals from South Africa and allowed the identification of ancestry-specific disease risk alleles associated with TB susceptibility that have previously been missed.

## Introduction

Pulmonary tuberculosis (TB), caused by the *bacillus Mycobacterium tuberculosis (M.tb)*, is a complex disease which affects populations disproportionately and results from a multifactorial interaction between host and pathogen (Yim and Selvaraj, 2010). It is often said that approximately 5–10% of infected individuals (±3 billion people worldwide) will go on to develop active TB whilst the majority will remain asymptomatic (Bañuls et al., 2015; El Kamel et al., 2015; Chaw et al., 2020). According to the World Health Organization (WHO), an estimated 10 million TB cases and 1.5 million deaths were reported in 2019 (WHO, 2019). TB therefore remains a global health burden and is of particular concern in low- to middle-income countries where a generally higher incidence rate (615 per 100 000 in South Africa) occurs, together with the limitations of currently available therapies and vaccines (Bao et al., 2016; WHO | Global tuberculosis report 2019, 2019). Numerous genetic and heritability studies have established the role of host genetic factors in susceptibility to TB (Rudko et al., 2016; Kinnear et al., 2017; Cai et al., 2019; Luo et al., 2019), but with minimal overlap between populations from various geographical regions (Thye et al., 2010; Oki et al., 2011; Mahasirimongkol et al., 2012; Png et al., 2012; Thye et al., 2012; Chimusa et al., 2014, 2014; Curtis et al., 2015; Schurz et al., 2015; Grant et al., 2016; Sobota et al., 2016; Uren et al., 2017a; Omae et al., 2017; Qi et al., 2017; Zheng et al., 2018). The variation observed between populations from diverse geographic regions indicates possible ancestry-specific differences that contribute to the host genetic variability observed in TB genome-wide association studies (GWAS) (van Helden et al., 2006; Chimusa et al., 2014; Schurz et al., 2019a; Cai et al., 2019).

Previous investigations into southern African history and population structure elucidated indigenous KhoeSan ancestry in the region, in addition to populations being multi-way admixed due to multiple inter-and intra-continental migrations (de Wit et al., 2010; Quintana-Murci et al., 2010; Uren et al., 2017b). This population history has resulted in admixture from indigenous KhoeSan, Bantu-speaking African, European, Southeast Asian and East Asian populations (de Wit et al., 2010; Quintana-Murci et al., 2010; Uren et al., 2016). Ancestral populations contributed linked alleles (haplotype blocks) resulting in a mosaic of phenotypic consequences. This admixture can be leveraged to identify associations between various TB phenotypes and genomic regions harbouring variants with highly differentiated allele frequencies among ancestral populations, known as admixture mapping (Wang et al., 2020). Hence, the unique and complex admixed individuals from southern Africa, harbouring genomic contributions from ancestral populations with differing historical disease burden, present an opportunity to investigate ancestry-specific disease risk alleles associated with TB susceptibility (Shriner, 2013; Wang et al., 2020).

Previous admixture mapping and association studies investigating TB susceptibility loci in South Africa were restricted by a low number of controls, small reference population sample size and low SNP density (de Wit et al., 2010; Chimusa et al., 2014; Daya et al., 2014b, 2014a). With the recent adaption of computational algorithms to better suit multi-way admixed populations, a more suitable, high-density genotyping platform and the availability of large scale, population-specific datasets, we aimed to perform an updated scan for variants associated with TB using local ancestry adjusted allelic (LAAA) association models.

## Materials and Methods

### Study Population and Ethics Approval

A total of 413 pulmonary TB cases and 407 healthy controls were recruited from the metropolitan area of Cape Town in the Western Cape Province, South Africa. The population from this area was elected due to the high incidence of TB as well as the equal socio-economic status and low prevalence of HIV at the time of sampling (Rossouw et al., 2003; Möller et al., 2009; Gallant et al., 2010). Furthermore, TB cases and controls were sampled from the same area, therefore socio-economic status is unlikely to be a confounding factor as previously determined by Chimusa et al. (2014). TB cases were distinguished through bacteriological confirmation (culture positive and/or smear positive). Healthy controls had no previous history of TB. However, 80% of individuals above 15 years of age in this area were estimated to have been exposed to *M.tb*, and could therefore be regarded as latently infected (Gallant et al., 2010). If study participants were under the age of 18 or were HIV-positive, they were excluded from the analysis.

Written informed consent was obtained from all study participants before recruitment and blood collection. Sample collection (protocol number 95/072) and this study (S20/02/041) were both approved by the Health Research Ethics Committee of the Faculty of Health Sciences (HREC), Stellenbosch University. The research was conducted according to the principles expressed in the Declaration of Helsinki (2013).

### Genotyping, Data Merging and Quality Control

Genotype data on the case-control cohort was generated using the Illumina (Illumina, CA, United States) multi-ethnic genotyping array (MEGA) comprising ∼1.7 million markers (Schurz et al., 2019b). The Sanger Imputation Server (SIS) (https://imputation.sanger.ac.uk) and the African Genome Resource (AGR) reference panel (Gurdasani et al., 2015) was utilised for the imputation of missing genotypes. The imputed data was subjected to iterative quality control as previously described by Schurz et al. (2019b). Thereafter, the data from the admixed individuals were merged with the respective appropriate source populations (summarised in Table 1) using PLINK v2.0 (https://www.cog-genomics.org/plink/2.0/) (Purcell et al., 2007) in order to generate input files required for global and local ancestry inference.

**TABLE 1:** Ancestral populations included in analysis.

Population	*n*	Source
European (British)	60	1000G phase 3
African (Luhya)	50	1000G phase 3
East Asian (Chinese)	36	1000G phase 3
KhoeSan (Nama)	44	European Genome-Phenome archive- https://ega-archive.org/
South East Asian (Malay)	40	Wong et al. (2013)

After merging of admixed and source ancestral populations, all individuals missing more than 10% genotypes were removed, SNPs with more than 3% missing data were excluded and a Hardy-Weinberg equilibrium (HWE) filter was used in controls (threshold < 0.01). The data was screened for relatedness using the software KING (Manichaikul et al., 2010) and individuals up to second degree relatedness were subsequently removed. Variants with a minor allele frequency (MAF) below 1% were removed. The final dataset after quality control and data filtering consisted of 392 TB cases and 346 controls in addition to 289 ancestral individuals. A total of 4,249,442 variants passed quality control and filtering parameters.

### Global Ancestry Inference

ADMIXTURE was used to investigate the population substructure amongst our cohort, as well as to determine the correct number of contributing ancestries (Alexander and Lange, 2011; Zhou et al., 2011). This is a model-based approach to estimate individual ancestry coefficients of an individual’s genome from *k* ancestral populations and corresponding ancestral genotype frequencies through cross validation. For the purpose of computational efficiency, redundant single-nucleotide polymorphisms (SNPs) were removed and only tagging SNPs representative of the genetic haplotype blocks remained. Therefore, each SNP that has a linkage disequilibrium (LD) *r* ^2^ of >0.1 within a 50-SNP sliding window (advanced by 10 SNPs at a time) was removed. A total of 261,694 autosomal markers after LD pruning and 820 individuals (413 cases and 407 controls) were used to infer ancestry in an unsupervised manner for k = 3–10 (5 iterations). All 820 individuals were grouped into running groups of equal size together with 289 ancestral populations whilst inferring global ancestry proportions. Related individuals were included in separate running groups. Running groups were created to ensure an equal number of reference populations and admixed populations whilst removing relatedness as a confounding factor during global ancestry assignment. After determining the correct *k* number of contributing ancestries through cross validation, the software RFMix was used to infer global ancestry proportions for downstream statistical analysis, since ADMIXTURE is not as accurate as haplotype-based analyses (Uren et al., 2020). The software PONG was used for visualisation of global ancestry proportions and amalgamation of multiple iterations into the major mode (Behr et al., 2016).

### Local Ancestry Inference

Local ancestry inference requires phasing of haplotypes prior to inferring local ancestry. The software program SHAPEIT2 (Delaneau et al., 2013; Delaneau and Marchini, 2014) (utilizing the HapMap Genetic map – GRCh37) was used to phase the merged dataset before inferring local ancestry for each position in the genome using RFMix (Maples et al., 2013). RFMix is 30X faster than other local ancestry inference software and is accurate in multi-way admixture scenarios (Maples et al., 2013; Uren et al., 2020). Default parameters were used, except for the number of generations since admixture, which was set to 15, consistent with previous studies (Uren et al., 2016). Both global and local ancestry was inferred for 1,027 individuals (392 TB cases, 346 controls and 289 ancestral individuals) and 4,249,442 autosomal SNPs.

### Statistical Analysis

A Local Ancestry Adjusted Allelic (LAAA) model, first described by Duan et al. (2018), was used to investigate if there are allelic, ancestry-specific or ancestry-specific allelic associations with TB susceptibility in an admixed South African population (Duan et al., 2018). Dosage files were compiled at each locus as a biallelic state and were calculated as 0, 1 or 2 copies of a specific ancestry at any locus along the genome. Separate regression models for each ancestral group were fitted to investigate which ancestral population(s) drive the association between TB status and local ancestry at each locus. Genome-wide admixture proportions obtained from RFMix were included in all regression models to account for population structure. The smallest ancestry proportion (East Asian) was excluded as covariate to avoid complete separation of data. Therefore, four ancestral components (KhoeSan, African, European, and Southeast Asian) were included as covariates in association testing, together with age and gender. The number of alternate alleles (not the reference alleles) were counted, as these are more likely to be ancestry-specific. A total of 738 unrelated individuals (392 TB cases and 346 controls) and 4,249,442 autosomal markers were included in this analysis. The *glm()* function in R was used for logistic regression association testing.

The following four regression models were tested simultaneously to detect the source (allelic, ancestry or both ancestry-allelic effect) of the association signals observed:

1. Global ancestry proportions were included as covariates and thus represents the null model. This test is regularly used in GWAS to investigate whether an additive allelic dose affect exists on the phenotype, not considering local ancestry (Homozygous for the reference allele = 0; Heterozygous = 1; Homozygous for the alternate allele = 2).
2. Local ancestry expressed in terms of the number of copies of a specific ancestry (Ancestry of interest = 1; Other ancestries = 0) at a locus were included as covariates. This model is often utilised to conduct admixture mapping studies to elucidate ancestry effects of variants which showcases frequency disparities across ancestral populations (Homozygous for other ancestry = 0; Heterozygous = 1; Homozygous for ancestry of interest = 2).
3. Minor allelic effects were used in an additive manner and were included as covariates whilst still adjusting for local ancestry. Therefore, jointly testing for model 1 + 2.
4. This model utilises the ancestry-specific minor alleles at a locus, thus the minor alleles together with the corresponding ancestry of the minor allele were included as covariates (Minor allele and ancestry not on the same haplotypes = 0; Minor allele and ancestry are on the same haplotype = 1). This model is an extension to the allelic (3) and local ancestry (2) model by modelling the combination of the minor allele present at a specific locus and the ancestry of the specific allele at that genomic locus. (Both minor allele and ancestry not on the same haplotype = 0; Heterozygote (only one haplotype has both minor allele and ancestry on the same haplotype = 1; Both minor allele and ancestry on the same haplotype = 2).

Since the true underlying causal variants as well as the LD between the marker under study are unknown, modelling all three terms simultaneously is the most effective approach to elucidate causal variants in an admixed cohort with minimal power loss (Duan et al., 2018). Therefore, we can determine if a specific minor allele, ancestry or both a minor allelic and ancestry co-occurs with TB status more often than would be expected by chance.

The development of power and sample size analysis tools for mapping ancestry-specific effects are lacking. The power to detect significant associations depends greatly on the proportion of admixture, differences in effect sizes between diverse ancestries and differences in the allele risk frequencies among ancestral populations. It is noteworthy to highlight that this information will vary for each admixture scenario. Nonetheless, it remains critical to conduct some sort of power calculation to ensure the reliability of elucidating ancestry-specific genomic regions amongst admixed individuals. Hence, we conducted a priori power analysis in order to ensure the reliability of results given our samples size using G*Power (Faul et al., 2007, 2009).

To account for the multiple testing burden, the R package *STEAM* (Significance Threshold Estimation for Admixture Mapping) (Grinde et al., 2019) was used to estimate the genome-wide significance threshold. *STEAM* is specifically designed to estimate genome-wide significance thresholds for admixture mapping studies given the admixture proportions and number of generations since admixture. We quantified the degree of inflation by generating a Quantile-Quantile plot of the residuals.

## Results

### Global Ancestry Inference

**FIGURE 1:**
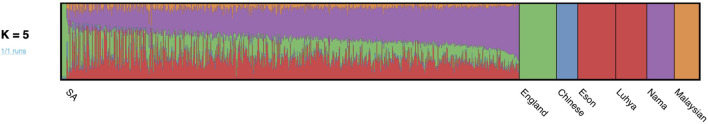
Genome-wide ancestral proportions of all SA individuals, with the ancestry proportion of each individual plotted vertically.

After close inspection of global ancestry proportions generated using ADMIXTURE, the *k* number of contributing ancestries was determined to be k = 5, since this was the lowest k-value through cross validation (Supplementary Table S1). Since haplotype-based admixture software is more accurate at global ancestry inference, ancestry proportions (genome-wide ancestral contributions) were inferred for all individuals using RFMix (Uren et al., 2020). Figure 1 represents the global ancestry proportions plotted vertically for each admixed individual and contributing ancestral populations using RFMix (k = 5). It is evident from the global ancestry inference that the cohort is a complex five-way admixed group, with ancestral contributions from the indigenous KhoeSan (∼35–40%), Bantu-speaking Africans (∼27–30%), Europeans (∼20%), Southeast Asians (∼7–8%) and East Asians (∼5%). Furthermore, extensive genetic heterogeneity can be observed, since genome-wide proportions differ vastly between individuals.

### Local Ancestry Inference

**FIGURE 2:**
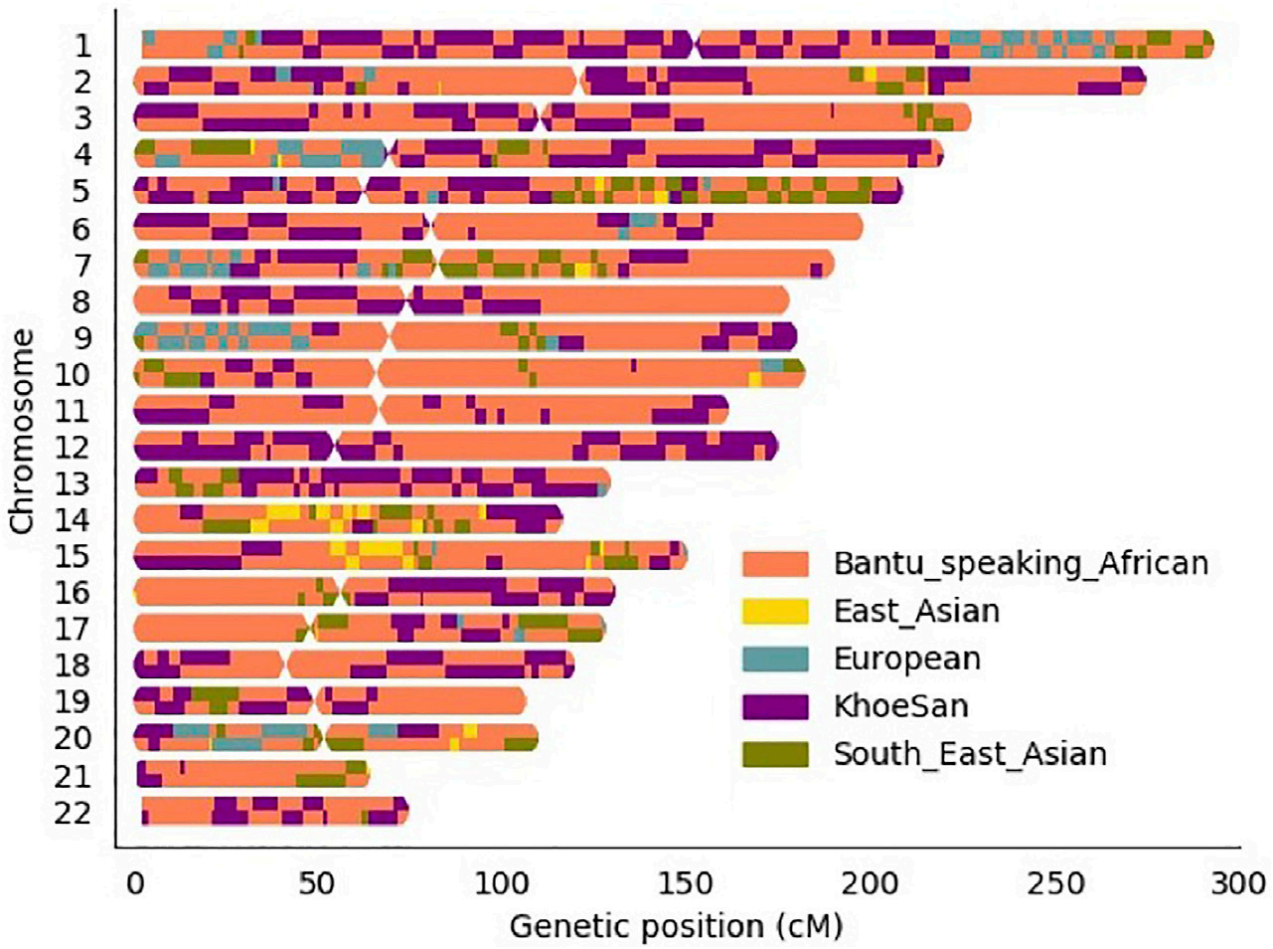
Karyogram of one admixed SA individual.

Local ancestry was estimated for all individuals and visually observed with karyograms. As shown in Figure 2, admixture between geographically distinct populations creates complicated ancestral-and admixture induced LD blocks. Figure 2 represents a single five-way admixed individual. Since not all individuals will harbour the same number and length of ancestry segments, it is necessary to accurately infer local ancestry in every individual at each genomic locus.

### Local Ancestry Allelic Adjusted Association Analysis

A total of 4,249,442 autosomal markers and 738 unrelated individuals (392 TB cases and 346 controls) were included in logistic regression models to assess whether any loci were significantly associated with TB status (adjusting for gender, age, and global ancestry proportions inferred by RFMix). More information regarding the distribution of age, gender and ancestry proportions of the cohort can be found in the Supplementary Figures S1–S3 and Supplementary Table S2. LAAA models were successfully conducted for all five ancestries present in this highly complex admixed cohort.

**FIGURE 3:**
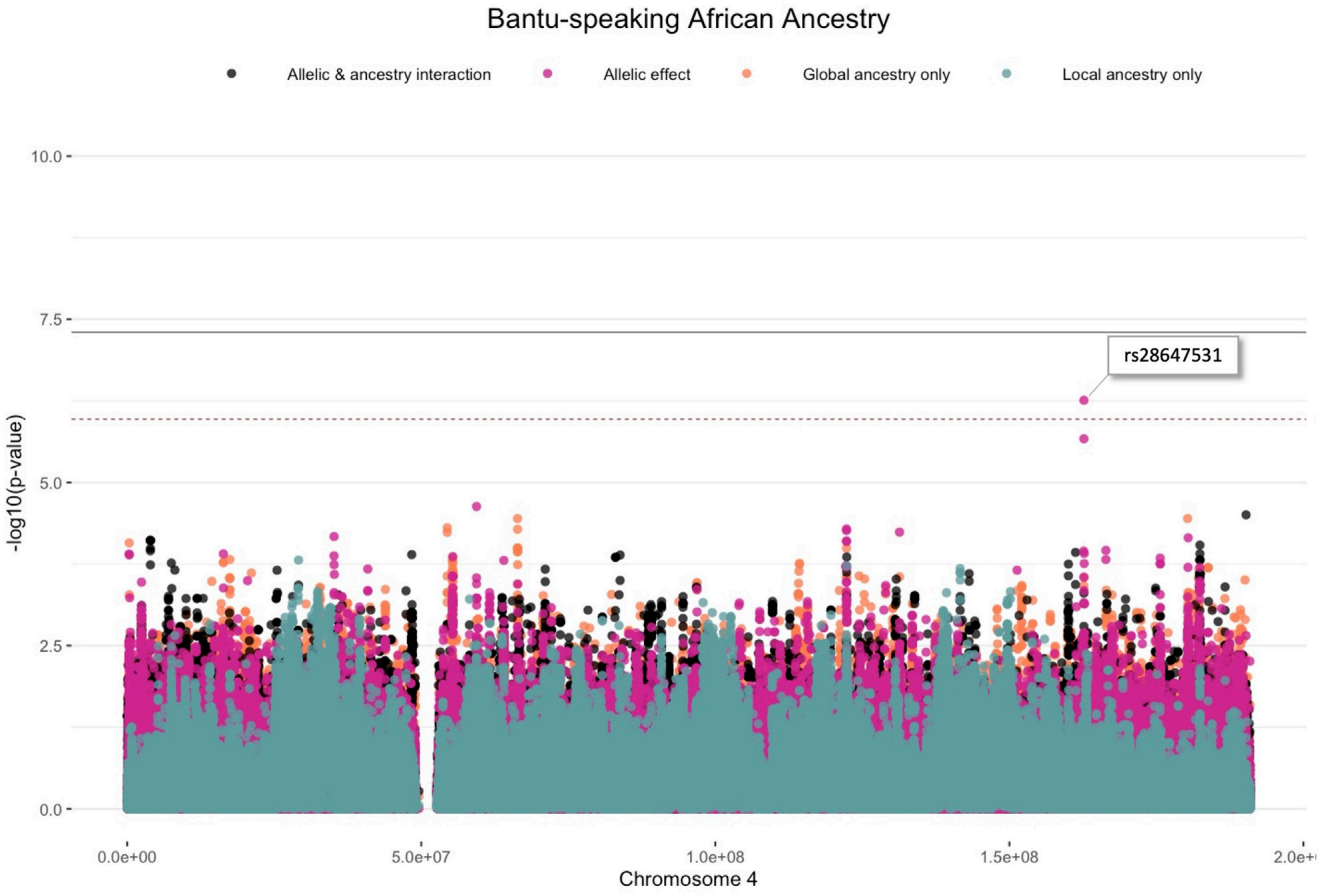
Log transformation of association signals (*p*-value < 1.078 × 10^−6^) obtained for Bantu-speaking African ancestry whilst using the allelic model whilst adjusting for local ancestry on chromosome 4. The dashed red line represents the significant threshold for admixture mapping calculated with the software *STEAM* and the black solid line represents the genome-wide significant threshold of 5 × 10^−8^. The four different models are represented in orange (global ancestry only), blue (local ancestry effect), pink (minor allelic effect only) and black (both minor allelic and ancestry effects).

One variant (rs28647531) was significantly associated with TB status (*p*-value < 1.078 × 10^−6^) due to an allelic SNP effect (G allele; 0.204 frequency) whilst adjusting for Bantu-speaking African local ancestry on chromosome 4 (OR = 3.065, *p*-value = 5.518 × 10^−7^) (Figure 3). This variant is an intronic variant with a gene consequence on Follistatin-related protein (*FSTL5*), which is a protein coding gene involved in calcium ion binding. No restrictions on the analysis or inflation of results were observed as indicated by the Quantile-Quantile plot (Supplementary Figure S4). Although no other variants passed the significance threshold, multiple lead variants (*p*-value < 1 × 10^−5^) were identified. Furthermore, it is clear from our results that multiple distinct lead variants were identified for each ancestry.

The lead variants identified using only the global ancestry as covariates (model 1), are summarised in Supplementary Table S3. One lead variant (rs38672118) is near the protein coding gene, *CUL2* (Cullin-2), located on chromosome 10. The lead variants identified by conducting admixture mapping (model 2), are summarised in Supplementary Table S4. Only one ancestry (European) identified a local ancestry peak on chromosome 15 (Supplementary Figure S5). The lead variants identified utilising the allelic model adjusting for local ancestry (model 3), are summarised in Table 2. The lead variants identified by the LAAA model (model 4) are summarised in Table 3. It is noteworthy that both the allelic model adjusting for local ancestry (model 3), and the LAAA model (model 4) captured association signals not previously observed for this cohort.

**TABLE 2:** Summary statistics of the top results (*p*-value < 1 × 10^−5^) whilst utilising the Additive allelic model whilst adjusting for local ancestry.

Chr	Position	rsID	Ref	Alt	Altfreq	OR	SE	*p*-value	Ancestry	Location	Gene
4	153960368	rs1024148	T	G	0.463	1.589	0.126	9.948e-06	European	None	None
13	24752695	rs7325698	T	C	0.310	1.363	0.150	7.721e-06	European	Intronic	*SPATA13*
13	24753449	rs2862243	A	G	0.263	1.300	0.174	3.811e-06	European	Intronic	*SPATA13*
13	50909771	rs67217502	T	C	0.103	1.108	0.225	6.474e-06	European	Intronic	*DLEU1*
13	50913874	rs12853498	A	T	0.103	1.108	0.225	6.474e-06	European	Intronic	*DLEU1*
13	50915280	rs17074141	C	T	0.103	1.108	0.225	6.474e-06	European	Intronic	*DLEU1*
13	50920890	rs17363026	T	C	0.104	1.110	0.225	4.129e-06	European	Intronic	*DLEU1*
13	50922773	rs17074143	T	A	0.099	1.104	0.230	5.059e-06	European	Intronic	*DLEU1*
13	50925317	rs34712361	T	C	0.098	1.103	0.230	7.398e-06	European	Intronic	*DLEU1*
13	50925565	rs67964536	C	T	0.098	1.103	0.230	7.398e-06	European	Intronic	*DLEU1*
13	50926076	rs79714483	A	G	0.098	1.103	0.230	7.398e-06	European	Intronic	*DLEU1*
14	48325261	rs447600	T	A	0.459	1.582	0.123	4.350e-06	European	None	None
2	52241352	rs2883609	C	G	0.318	1.374	0.122	9.361e-06	East Asian	nRNA_intronic	*AC007682.1*
2	180940603	rs13411512	T	C	0.274	1.315	0.130	7.428e-06	East Asian	Intronic	*CWC22*
12	9388842	ss1388098326	C	T	0.124	1.132	0.176	9.961e-06	East Asian	nRNA_intronic	*A2MP1*
14	48325261	rs447600	T	A	0.459	1.582	0.107	5.988e-06	East Asian	Intergenic	*RP11-476J6.1*
22	46046477	rs134850	A	G	0.223	1.250	0.131	7.042e-06	East Asian	Intergenic	*ATXN10*
1	151185502	rs4971014	A	G	0.187	1.206	0.150	3.279e-06	SouthEast Asian	Intronic	*PIP5K1A*
2	52241352	rs2883609	C	G	0.318	1.374	0.126	9.022e-06	SouthEast Asian	ncRNA_intronic	*AC007682.1*
2	180940603	rs13411512	T	C	0.274	1.315	0.132	5.645e-06	SouthEast Asian	Intergenic	*CWC22*
8	126754436	rs12547413	T	C	0.135	1.144	0.170	8.440e-06	SouthEast Asian	Intronic	*CLU2*
10	35527543	rs3867218	C	T	0.513	1.670	0.116	3.194e-06	SouthEast Asian	Intergenic	*RNU6794P*
14	90278083	rs10137384	T	C	0.121	1.129	0.192	8.522e-06	SouthEast Asian	ncRNA_intronic	*RP1133N16.3*
21	43759441	rs692544	C	T	0.508	1.662	0.115	4.414e-06	SouthEast Asian	Intergenic	*TFF2*
22	46036079	rs1894617	G	C	0.235	1.265	0.129	7.610e-06	SouthEast Asian	Intergenic	*RNU6794P*
22	46046477	rs134850	A	G	0.223	1.250	0.131	6.868e-06	SouthEast Asian	Intergenic	*ATXN10*
2	143729878	rs10928161	C	T	0.129	1.137	0.204	7.369e-06	KhoeSan	Intronic	*KYNU*
2	143730019	rs16855223	G	A	0.130	1.139	0.204	6.268e-06	KhoeSan	Intronic	*KYNU*
2	143731496	rs35991933	A	T	0.129	1.137	0.204	7.369e-06	KhoeSan	Intronic	*KYNU*
2	143731661	rs34891373	T	A	0.129	1.137	0.204	7.369e-06	KhoeSan	Intronic	*KYNU*
2	143737201	rs11904225	G	A	0.146	1.157	0.194	1.374e-06	KhoeSan	Intronic	*KYNU*
2	143742532	rs10496933	G	A	0.129	1.138	0.204	5.676e-06	KhoeSan	Intronic	*KYNU*
2	143743246	rs12463750	G	A	0.146	1.157	0.194	1.374e-06	KhoeSan	Intronic	*KYNU*
2	180940603	rs13411512	T	C	0.274	1.315	0.164	8.617e-06	KhoeSan	Intronic	*KYNU*
4	54413304	rs4864469	T	C	0.067	1.070	0.247	8.725e-06	KhoeSan	ncRNA_intronic	*FIP1L1*
4	114309839	rs6533681	C	T	0.356	1.427	0.157	4.856e-06	KhoeSan	Intergenic	*ANK2*
5	81172726	rs62368165	G	A	0.401	1.494	0.180	9.898e-06	KhoeSan	Intergenic	*SHFM1P1*
6	7328023	rs145663084	T	C	0.113	1.120	0.211	4.122e-06	KhoeSan	ncRNA_exonic	*PRSS23*
11	86632570	rs612410	T	C	0.337	1.400	0.158	8.791e-06	KhoeSan	Intronic	*PRSS23*
11	86641079	rs10792884	A	G	0.361	1.435	0.160	9.751e-06	KhoeSan	Intronic	*PRSS23*
11	86641484	rs7940935	C	T	0.363	1.437	0.160	9.775e-06	KhoeSan	Intronic	*PRSS23*
11	86642522	rs10792886	G	A	0.363	1.437	0.160	8.430e-06	KhoeSan	Intronic	*PRSS23*
11	86643022	rs7948323	C	A	0.364	1.439	0.160	8.311e-06	KhoeSan	Intronic	*PRSS23*
11	86643351	rs10751145	A	G	0.364	1.439	0.160	8.311e-06	KhoeSan	Intronic	*PRSS23*
11	86644159	rs10792887	G	A	0.364	1.439	0.160	8.311e-06	KhoeSan	Intronic	*PRSS23*
11	86644244	rs10792888	A	C	0.364	1.439	0.160	8.311e-06	KhoeSan	Intronic	*PRSS23*
11	86644300	rs7484279	C	T	0.364	1.439	0.160	8.311e-06	KhoeSan	Intronic	*PRSS23*
11	86644938	rs3740665	C	T	0.365	1.440	0.160	8.889e-06	KhoeSan	Intronic	*PRSS23*
11	86645157	rs10898560	A	G	0.366	1.442	0.160	9.506e-06	KhoeSan	Intronic	*PRSS23*
11	86645214	rs1902425	C	T	0.365	1.440	0.160	8.889e-06	KhoeSan	Intronic	*PRSS23*
11	124196782	rs676720	C	A	0.239	1.271	0.162	8.973e-06	KhoeSan	Intergenic	*OR8B7P*
11	124199570	rs7119360	A	G	0.239	1.271	0.162	8.973e-06	KhoeSan	Intergenic	*OR8B7P*
12	18844727	rs10841067	T	C	0.050	1.052	0.292	4.802e-06	KhoeSan	Intronic variant	*PLCZ1*
12	18845754	rs1973289	C	T	0.054	1.056	0.282	1.710e-06	KhoeSan	ncRNA_exonic	*PLCZ1*
12	18846108	rs2900416	A	G	0.054	1.056	0.282	1.710e-06	KhoeSan	ncRNA_exonic	*PLCZ1*
15	39513293	rs7176317	C	G	0.516	1.675	0.127	2.405e-06	KhoeSan	ncRNA_exonic	*RP11-624L4.1*
4	162663106	rs10517752	G	A	0.199	1.221	0.222	2.142e-06	African	Intronic	*FSTL5*
4	162663775	rs28647531	A	G	0.204	1.226	0.224	5.518e-07	African	Intronic	*FSTL5*
17	75030582	rs11077888	G	A	0.470	1.600	0.142	3.400e-06	African	Intergenic	*AC015815.5*
18	41342728	rs11659620	T	C	0.081	1.084	0.251	5.876e-06	African	Intergenic	*RNU6443P*
18	41351686	rs1822027	T	G	0.081	1.084	0.251	5.876e-06	African	Intergenic	*RNU6443P*
18	41352221	rs35810759	A	G	0.081	1.084	0.251	5.876e-06	African	Intergenic	*RNU6443P*
21	43759441	rs692544	C	T	0.508	1.662	0.136	1.094e-06	African	Intergenic	*TFF2*

**TABLE 3:** Summary statistics of the top results (*p*-value < 1 × 10^−5^) whilst utilising the Local Ancestry Adjusted Allelic (LAAA) model.

Chr	Position	rsID	Ref	Alt	Altfreq	OR	SE	*p*-value	Ancestry	Location	Gene
1	208027696	rs61821315	C	T	0.132	1.141	0.356	8.804e-06	African	None	None
1	208029947	rs7550821	C	T	0.132	1.141	0.358	4.771e-06	African	None	None
1	208030856	rs7551724	C	T	0.131	1.140	0.359	8.065e-06	African	None	None
2	59343477	rs17049931	C	T	0.148	1.160	0.318	5.919e-06	African	None	None
2	172987232	rs7583008	T	C	0.230	1.259	0.318	9.628e-06	African	None	None
2	172987357	rs7569224	G	C	0.230	1.259	0.318	9.628e-06	African	None	None
3	22648301	rs1449916	T	C	0.394	1.483	0.307	3.665e-06	African	None	None
3	104923287	rs13061116	G	A	0.216	1.241	0.357	5.134e-06	African	None	None
3	104923579	rs1525840	T	C	0.216	1.241	0.357	6.076e-06	African	None	None
3	104924774	rs11923672	A	T	0.216	1.241	0.357	6.076e-06	African	None	None
3	104924866	rs11926446	G	A	0.216	1.241	0.357	5.134e-06	African	None	None
3	104929569	rs9834777	T	C	0.217	1.242	0.357	5.161e-06	African	None	None
5	31584670	rs10940959	C	A	0.123	1.131	0.380	5.596e-06	African	None	None
9	73895875	rs7037178	T	G	0.459	1.582	0.235	8.903e-06	African	Intronic	*TRPM3*
9	73899145	rs1504387	T	C	0.461	1.586	0.234	3.558e-06	African	Intronic	*TRPM3*
11	126163124	rs609634	T	C	0.261	1.298	0.255	7.570e-06	African	Intronic	*TIRAP*
18	65322790	rs1444107	T	A	0.082	1.085	0.446	6.644e-06	African	Intronic	*DSEL-AS1*
18	65323846	rs2448767	A	G	0.082	1.085	0.446	6.644e-06	African	Intronic	*DSEL-AS1*
18	65324070	rs2448766	A	G	0.082	1.085	0.446	6.644e-06	African	Intronic	*DSEL-AS1*
2	183351225	rs1594304	T	C	0.421	1.523	0.253	3.557e-06	Khoesan	Intronic	*PDE1A*
5	26027283	rs12659706	C	T	0.337	1.401	0.340	9.503e-06	Khoesan	None	None
6	9576203	rs4715321	G	T	0.406	1.501	0.288	7.051e-06	Khoesan	None	None
5	142454386	rs13340374	C	T	0.061	1.063	0.532	3.421e-06	European	Intronic	*ARHGAP26*
9	119475712	rs72763937	C	T	0.184	1.202	0.320	8.548e-06	European	Intronic	*ASTN2*
11	126163124	rs609634	T	C	0.261	1.298	0.311	9.909e-06	European	Intronic	*TIRAP*
20	36998495	rs11698149	T	C	0.064	1.066	0.520	7.203e-06	European	Intronic	*LBP*
9	73099454	AS	T	C	0.324	1.383	0.531	9.295e-06	SouthEast Asian	Intronic	*KLF9-DT*

## Discussion

We conducted local ancestry allelic adjusted association analysis in a multi-way admixed South African (SA) population to investigate whether ancestry-specific genetic regions are associated with TB susceptibility. Multi-way admixed populations allow the opportunity to simultaneously assess the association of TB status in multiple continental populations and elucidate possible ancestry-specific effects on TB susceptibility. Previous studies were confounded by the limited number of representative reference populations available to infer local ancestry and the use of the low-density Affymetrix gene chip array (∼500k markers) in the analyses. New, more representative ancestral populations and an increase in accuracy of several software tools facilitated the novel findings presented here.

Global ancestry deconvolution suggested a five-way admixed scenario for the study cohort. This is in accordance with previous studies (de Wit et al., 2010; Chimusa et al., 2014; Uren et al., 2016). This diverse admixture and associated regional heterogeneity are reflected in the karyograms generated via local ancestry inference (Figure 2). This scale of genetic heterogeneity suggests that no two individuals will harbour the same DNA segment from the same ancestral population, i.e., there is a high degree of locus-specific ancestry (Duan et al., 2018). The results presented here highlight that only including global ancestry proportions in the analysis is not sufficient to identify which ancestry is located on distinct chromosomal segments. The only lead variant (rs38672118) identified using the global ancestry-only model is near the protein coding gene, *CUL2*. Although the function of *CUL2* on *M.tb* clearance is still uncertain, *CUL2* forms an important part of the cullin-RING-based E3 ubiquitin-protein ligase complex and subsequently targets the ubiquitination of target proteins (Nguyen et al., 2017). The model used for admixture mapping (only utilising local ancestry) seems overconservative for complex multi-way admixed individuals, since only one admixture peak was close to the significance threshold for European ancestry (located on chromosome 15). This highlights the phenomenon of genetic heterogeneity where the presence of both admixture-induced LD blocks and haplotype LD blocks often results in missed association signals due to tagging SNPs being possibly located in different ancestral LD blocks (Duan et al., 2018).

**FIGURE 4:**
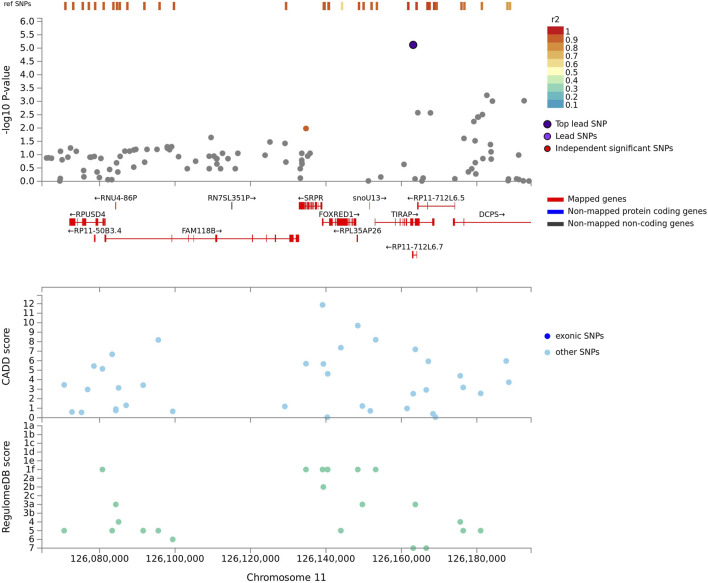
Regional plot indicating the nearest genes in linkage disequilibrium for the lead variant observed for Bantu-speaking African ancestry on chromosome 11, whilst utilising the LAAA model. SNPs not in linkage disequilibrium are coloured grey and the lead variant is indicated in purple. Mapped genes are coded in red.

One example of missing relevant associated variants in complex admixed populations, is the association signal obtained on chromosome 11q13 while adjusting for Bantu-speaking African- and European local ancestry. This lead variant indicated an association with the TIR Domain Containing Adaptor Protein (*TIRAP*) gene (Figure 4) and is involved in the toll-like receptor (TLR) 4 signalling pathway of the immune system via the TIR adaptor protein it codes for. *TIRAP* is a protein which identifies microbial pathogens trough TLRs as part of the initial innate immune response (Selvaraj et al., 2010). This acts via *IRAK2* and *TRAF-6*, leading to the activation of NF-kappa-B, MAPK1, MAPK3 and JNK, which is essential for cytokine secretion in order to mount an inflammatory response (Capparelli et al., 2013). Polymorphisms in the *TIRAP* gene were previously identified to be associated with TB susceptibility in a South Indian population (Selvaraj et al., 2010), as well as a Chinese population (Zhang et al., 2011). This suggests a possible role of the *TIRAP* gene in TB susceptibility via activation of TLRs in order to recognize several components of *M.tb* during active TB disease*.* The T allele of TLR4 (rs4986791) was found to be associated with an increased risk for an Asian subgroup in a meta-analysis investigating TLR variants and susceptibility to TB (Schurz et al., 2015). Additionally, chromosome 11p13 was also previously associated with African ancestry in a previous GWAS (Thye et al., 2012; Chimusa et al., 2014). If the allelic model was not used while adjusting for local ancestry, this lead variant located near the *TIRAP* gene would have been missed due to the tagging SNP being located on a different ancestral haplotype LD block. This underlines the importance of including the LAAA models in association studies investigating complex multi-way admixed individuals.

One variant (rs28647531) passed the significance threshold and is located on chromosome 4q22 using the allelic model adjusting for Bantu-speaking African local ancestry (Figure 3). This variant is an intronic variant and located near the *FSTL5* gene, which has not been associated with TB susceptibility previously. This gene is a coding protein and was previously associated with colorectal cancer and acute myeloid leukaemia (Lv et al., 2017). Previous investigations of TB susceptibility in a southern African cohort identified African-and KhoeSan ancestry to be associated with an increased risk for TB (Chimusa et al., 2014, 2014; Daya et al., 2014b). Likewise, previous association signals for TB susceptibility in Africans included the *WT1* gene located on chromosome 11p13 and locus 18q12 and polymorphisms in the *TLR8* genes (Thye et al., 2010, 2012; Chimusa et al., 2014). Although we did not validate these genes in our study, we did however elucidate a lead variant located on chromosome 18q12 for Bantu-speaking African ancestry whilst utilising the LAAA model, meaning both the minor allele and ancestry co-occurs in this region. A previously unmapped protein coding gene (*DSEL-AS1*) was identified to be in LD with a leading SNP located on chromosome 18q12 for Bantu-speaking African ancestry (Supplementary Figure S6). *DSEL-AS1* is a lncRNA gene and was previously associated with unipolar depression, asparagine levels, bipolar disorder, body mass index and gut microbiome levels (Shi et al., 2011; Rhee et al., 2013; Winham et al., 2014; Ishida et al., 2020), but no biological pathways or interactions were reported for this lncRNA.

Moreover, another lead variant was identified for Bantu-speaking African ancestry. Transient receptor potential cation channel subfamily Melastatin member 3 (*TRPM3*), located on chromosome 9, is a protein coding gene which belongs to the family of transient receptor potential (TRP) channels. *TRPM3* is a permeable non-selective cation gene channel (Zhao et al., 2020, 3). Therefore, this gene is essential for cellular calcium signalling and homeostasis. Previous GWAS indicated the potential role of *TRPM3* in the measurement of mean platelet volume and were previously discovered in mostly European individuals (Astle et al., 2016; Vuckovic et al., 2020). Another protein coding gene, Phosphodiesterase 1A (*PDE1A*), is involved in calcium signalling and was amongst the lead variants identified for KhoeSan ancestry located on chromosome 2q14 by the LAAA model. This gene forms part of the cyclic nucleotide phosphodiesterases, which plays a role in signal transduction by regulating intracellular cyclic nucleotide concentrations through hydrolysis of cAMP and/or cGMP to their respective nucleoside 5-prime monophosphates. Therefore, this gene is important for calmodulin binding and cGMP binding, as well as associated with urate measurement and glomerular filtration rate (Hellwege et al., 2019; Gill et al., 2021). Hence, there is evidence of the role of calcium ion channel activity in TB susceptibility, which includes the *FSTL5* gene and *TRPM3* gene for African ancestry, and the *PDE1A* gene for KhoeSan ancestry. *M.tb* modulates the levels and activity of key intracellular second messengers, such as calcium, to evade protective immune responses. Furthermore, calcium plays a crucial role in *M.tb* pathogenesis by activating differential transcription factors or mediating of the phagosome-lysosome fusion and cell survival (Sharma et al., 2016).

Our results demonstrate the benefit of simultaneously modelling allele, local ancestry, and ancestry-specific minor allelic effects when the admixed population under study exhibits extreme heterogeneity, since multiple distinct ancestry-specific genetic variants were identified for TB susceptibility that were previously missed by standard analyses. Thus, including an interaction term between the minor allele present and the corresponding ancestry of that minor allele can robustly identify ancestry-specific effects on disease phenotypes in a complex admixed population. It is important to mention that only variants that met certain quality control criteria during the imputation procedure were included in our analysis. Furthermore, minor alleles might have become evident after populations diverged, or have occurred in recent human history, and they are more likely to be ancestry-specific (Qin et al., 2019). The LAAA model first described by Duan et al. (2018) counts the number of reference alleles, whereas we counted the number of copies of the alternate alleles. Minor alleles might have become evident after populations diverged, or have occurred in recent human history, and they are more likely to be ancestry-specific (Qin et al., 2019). Therefore, allowing the detection of minor ancestry-specific allelic effects.

Currently there is no clear best practise for deriving the significance cut-off threshold for admixture mapping studies. Every admixture scenario is unique in terms of contributing ancestral source populations, density markers analysed and particularly generations since admixture occurred. Moreover, in the presence of correlated tests the Bonferroni correction for multiple testing burden is overconservative for admixture mapping studies and does not necessarily control for family-wise error rate control in association analysis (Grinde et al., 2019). For this reason, we used the method described by Grinde et al. (2019), which entails a test statistic simulation directly from the asymptotic distribution implemented in the R software package *STEAM.* It considers the number of contributing ancestral populations, number of generations since admixture occurred and the distribution of admixture proportions in the cohort of interest and permutes these factors 1,000 times to get a new cut-off for significance (Grinde et al., 2019).

A limitation of the current study is the small sample size and findings should be validated in additional larger cohorts from various ethnic groups. Given our sample size of 735 participants (392 TB cases and 346 controls), we have 95% chance to correctly rejecting the null hypothesis for large (>0.5) and medium effect sizes (>0.3). We do however lose power if the effect size is small (0,1–0,3) and any reported associations with a smaller effect size should therefore be interpreted with caution (Supplementary Figure S7). Furthermore, there is a possibility that the true effect could be smaller than 0.1 for ancestry-specific effects in five different continental populations, confounding the study power (Skotte et al., 2019). Since literature suggests that TB susceptibility is governed by numerous SNPs with small effect sizes, we may have missed true local ancestry effects (type 2 errors) due to our small sample size. To report on ancestry-specific susceptibility to TB in a multi-way admixed southern African population, we estimate that at least 5,568 participants are required to confidently identify markers with smaller effect sizes (0.1–0.3).

Future studies should also include *in silico* and *in vitro* validation. Moreover, progression to active TB might be explained by numerous variants having a small effect on disease outcome, or exceptionally rare variants (Schurz et al., 2015). Variants that are unique to different populations and at low frequency should also be interrogated in well-powered studies. In addition, the information on the infecting *M.tb* strain should also be included in association analysis, if possible, since it appears that *M.tb* co-evolved with humans (Brites and Gagneux, 2015) and that the interaction between host genes and *M.tb* lineage affects TB severity (Müller et al., 2021). The combination of the ancestral allele and older *M.tb* lineages, i.e., the genotype and lineage that co-existed historically, had the lowest average TB score (McHenry et al., 2020). According to the TB score system, individuals are ranked according to their relative risk of being infected with TB given certain diagnostic information. A TB score of more than 40 indicates that a TB diagnosis is highly likely, a score of 30–35 indicates a possible TB diagnosis and a score below 25 indicates an unlikely diagnosis (dos Santos et al., 2017). Thus, the host populations that were historically exposed to a specific lineage have a lower chance of disease. Similarly, the average TB score for the combinations of genotype and lineage that have not historically co-existed, were the highest (McHenry et al., 2020). Thus, the evolutionary history of both species should be considered together.

In conclusion, this is the first study to apply the LAAA model to a complex five-way admixed population from South Africa which exhibits extensive genetic heterogeneity. This was enabled by newly developed algorithms for local ancestry inference, updated reference panels to represent contributing ancestral populations and a more suitable genotyping platform for diverse populations worldwide. We have demonstrated that the LAAA model robustly captured the source of association signals in highly complex admixed individuals. The true underlying architecture at each locus is unknown for most southern African populations, indicating that careful consideration of both global-and local ancestry is required for successful complex-trait mapping. Furthermore, local ancestry information across the genome is likely to become relevant to determine whether a genetic variant is expected to be useful in precision medicine, specifically in admixed populations.

## Data Availability

The data analyzed in this study is subject to the following licenses/restrictions: No new genetic data was generated for this study however, summary statistics for the quality and accuracy assessment of the genetic data will be made available to researchers who meet the criteria for access after application to the Health Research Ethics Committee of Stellenbosch University. Requests to access these datasets should be directed to MM, marlom@sun.ac.za.
