# Supplementary material for: Local Ancestry Adjusted Allelic Association Analysis Robustly Captures Tuberculosis Susceptibility Loci
Source: Front Genet. 2021 Oct 15;12:716558. doi: 10.3389/fgene.2021.716558 (PMC8554120; doi:10.3389/fgene.2021.716558)
Supplement: Supplementary file 1 [file DataSheet1.docx]

Supplementary Material

**Supplementary table 1.** Cross validation values for each number of *k* (3-10).

| CV error (k=3): 0.24532 |
| --- |
| CV error (k=4): 0.24305 |
| **CV error (k=5): 0.23914** |
| CV error (k=6): 0.23947 |
| CV error (k=7): 0.24037 |
| CV error (k=8): 0.24177 |
| CV error (k=9): 0.23926 |
| CV error (k=10): 0.23946 |

**Supplementary table 3.** Summary statistics of the top results (p-value < 1×10^-5^) whilst utilising the Global Ancestry only model.

| **Chr** | **Position** | **rsID** | **Ref** | **Alt** | **Altfreq** | **OR** | **SE** | **P-value** | **Ancestry** | **Location** | **Gene** |
| --- | --- | --- | --- | --- | --- | --- | --- | --- | --- | --- | --- |
| 1 | 151185502 | rs4971014 | A | G | 0.187 | 0.532 | 0.141 | 7.580e-06 | Null model | None | None |
| 2 | 180940603 | rs13411512 | T | C | 0.274 | 0.558 | 0.127 | 4.554e-06 | Null model | None | None |
| 5 | 12599348 | rs7449177 | T | C | 0.467 | 1.619 | 0.109 | 9.944e-06 | Null model | None | None |
| 10 | 35527543 | rs3867218 | C | T | 0.513 | 1.670 | 0.112 | 5.003e-06 | Null model | Intergenic variant | *CLU2* |
| 14 | 48325261 | rs447600 | T | A | 0.459 | 5.311 | 0.105 | 2.108e-06 | Null model | None | None |
| 14 | 48349054 | rs181301 | A | G | 0.416 | 1.627 | 0.109 | 7.380e-06 | Null model | None | None |
| 21 | 43759441 | rs692544 | C | T | 0.508 | 1.671 | 0.114 | 6.624e-06 | Null model | None | None |

**Supplementary table 3.** Summary statistics of the top results (p-value < 1×10^-5^) whilst utilising the Local Ancestry only model.

| **Chr** | **Position** | **rsID** | **Ref** | **Alt** | **Altfreq** | **OR** | **SE** | **P-value** | **Ancestry** | **Location** | **Gene** |
| --- | --- | --- | --- | --- | --- | --- | --- | --- | --- | --- | --- |
| 15 | 36810502 | rs12898382 | C | A | 0.296 | 0.813 | 0.180 | 6.300e-06 | European | None | None |
| 15 | 36811610 | rs8026282 | T | C | 0.297 | 0.811 | 0.180 | 6.647e-06 | European | None | None |
| 15 | 36811912 | rs8042225 | C | T | 0.290 | 0.830 | 0.180 | 4.047e-06 | European | None | None |
| 15 | 36812176 | rs12905532 | A | G | 0.290 | 0.834 | 0.180 | 3.599e-06 | European | None | None |
| 15 | 36812687 | rs969687 | T | C | 0.290 | 0.838 | 0.180 | 3.267e-06 | European | None | None |
| 15 | 36812906 | rs12102111 | A | G | 0.288 | 0.825 | 0.180 | 4.451e-06 | European | None | None |
| 15 | 36812913 | rs969686 | C | T | 0.288 | 0.825 | 0.180 | 4.451e-06 | European | None | None |
| 15 | 36812923 | rs969685 | T | C | 0.288 | 0.825 | 0.180 | 4.451e-06 | European | None | None |
| 15 | 36814981 | rs12438049 | A | C | 0.294 | 0.809 | 0.182 | 8.790e-06 | European | None | None |
| 15 | 36816133 | rs8029917 | T | C | 0.294 | 0.809 | 0.182 | 8.790e-06 | European | None | None |
| 15 | 36820330 | rs12899475 | G | A | 0.295 | 0.805 | 0.182 | 9.630e-06 | European | None | None |

**Supplementary figure 1.** Boxplot of ancestry proportions between males and females in TB cases and controls.

**Supplementary figure 2.** Boxplot of ancestry proportions between TB cases and controls.

**B**

**A**

**Supplementary figure 3. A)** Bar plot of proportions of females (Blue) and males (Orange) between TB cases and controls. **B)** Bar plot of age distributions between TB cases and controls.

**Supplementary Figure 4.**  Quantile-quantile plot of expected p-values and observed p-values for association signal obtained for Bantu-speaking African ancestry located on chromosome 4.


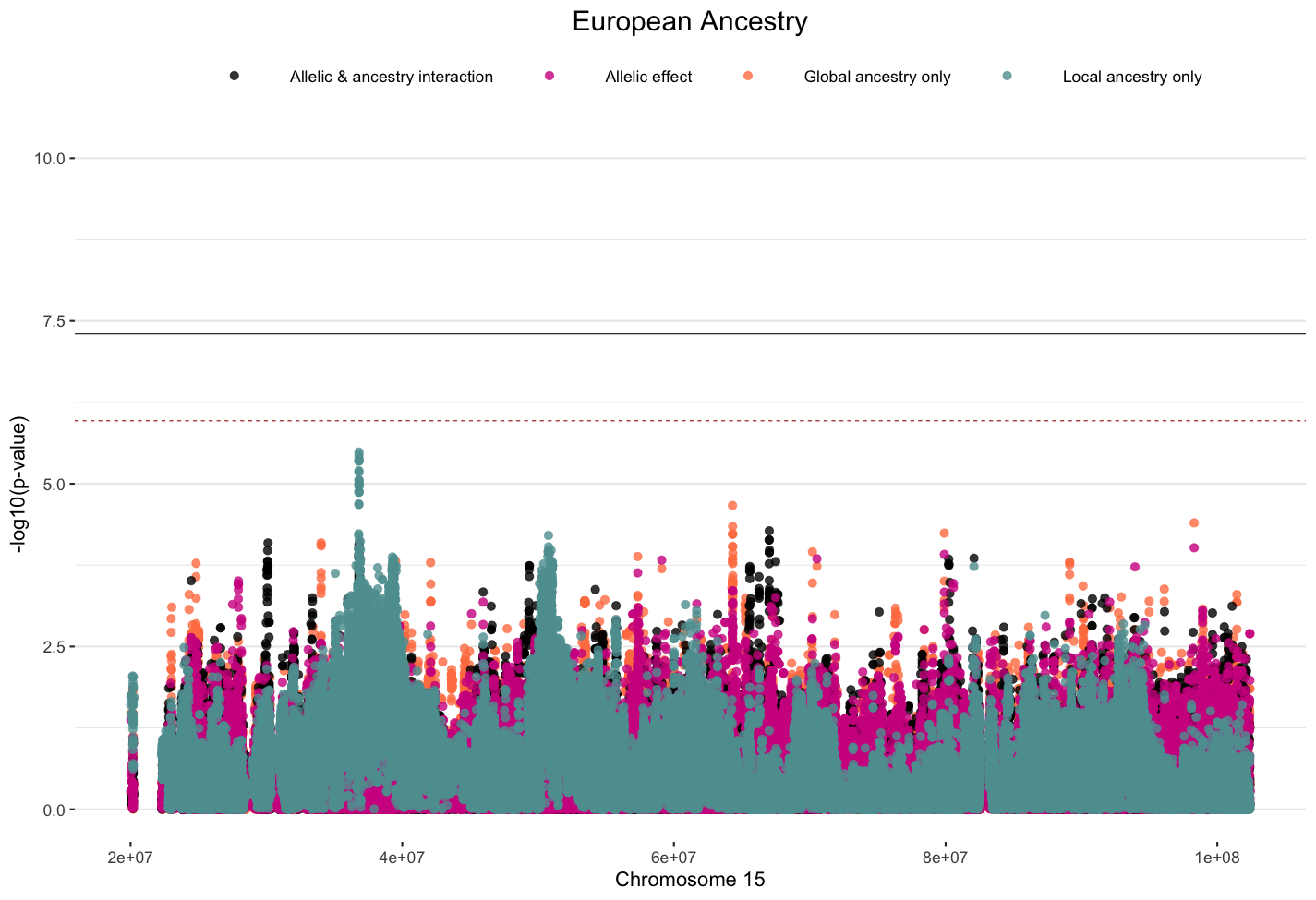


**Supplementary Figure 5.** Log transformation of association signals obtained for European ancestry on chromosome 15. The dashed red line represents the significant threshold for admixture mapping calculated with the software *STEAM* and the black solid line represents the genome-wide significant threshold of 5×10^-8^. The four different models are represented in orange (global ancestry only), blue (local ancestry effect), pink (minor allelic effect only) and black (both minor allelic and ancestry effects).


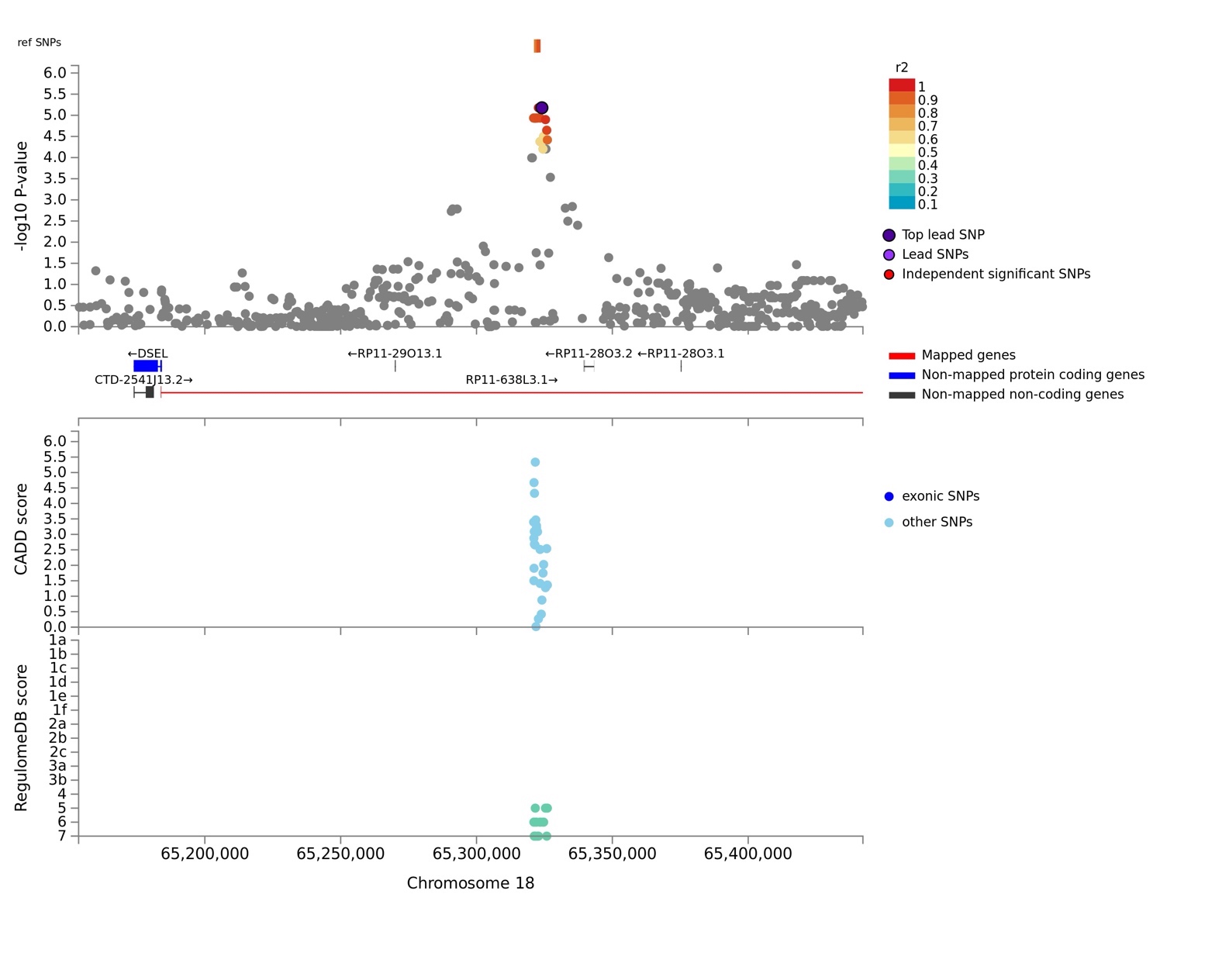
**Supplementary Figure 6.** Regional plot for leading variants in linkage disequilibrium on chromosome 18 for Bantu-speaking African ancestry, whilst utilising the LAAA model.


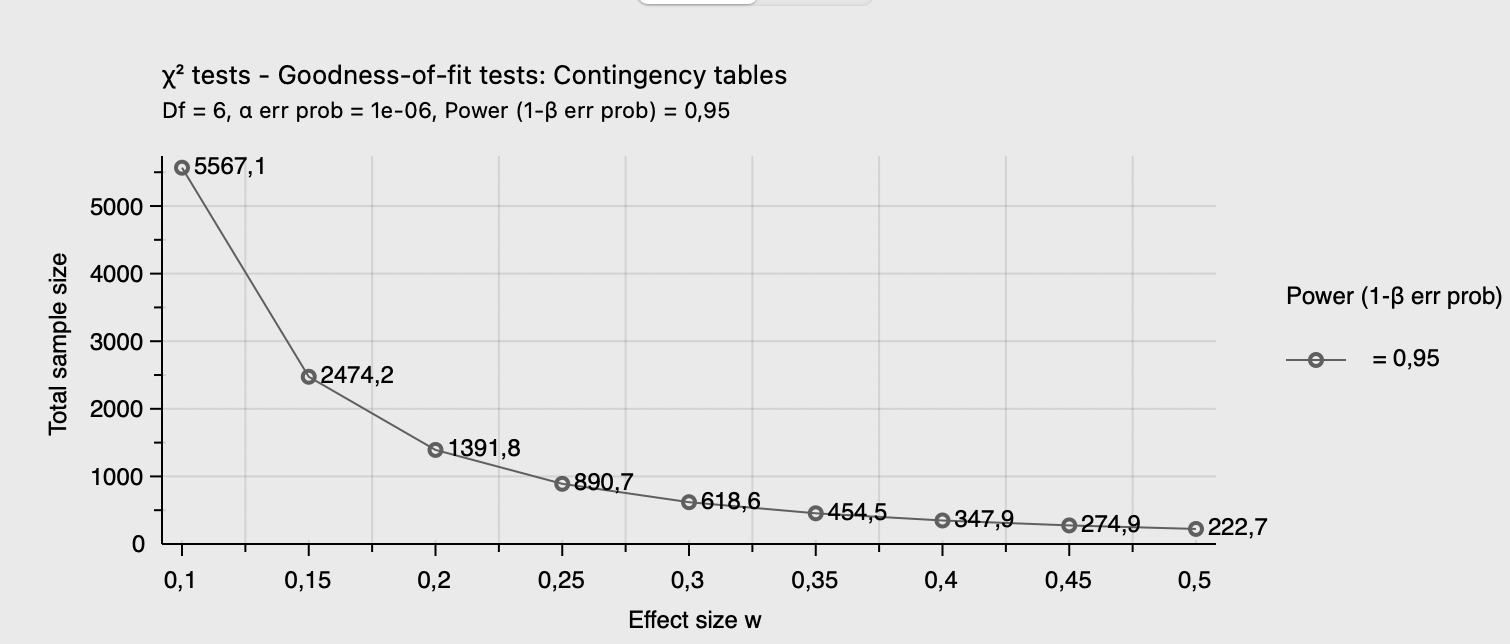


**Supplementary Figure 7.** Plot indicating the total samples size (y-axis) required for a range of effect sizes (x-axis) using the Chi-square goodness of fit test. An alpha value of 1e-06 was used, degrees of freedom= 6 and power was set at 95%.
